# Multidimensional Functional Phenotyping Based on Photoreceptor-Directed Temporal Contrast Sensitivity Defects in Inherited Retinal Diseases

**DOI:** 10.1167/iovs.66.4.25

**Published:** 2025-04-10

**Authors:** Cord Huchzermeyer, Katarina Stingl, Jan Kremers

**Affiliations:** 1 Department of Ophthalmology, University Hospital Erlangen, Erlangen, Germany; 2 Center for Ophthalmology, University Hospital, University of Tübingen, Tübingen, Germany

**Keywords:** occult macular dystrophy, retinitis pigmentosa, Stargardt's disease, silent substitution, temporal contrast sensitivity

## Abstract

**Purpose:**

To identify patterns of functional defects in perifoveal photoreceptor-directed temporal contrast sensitivities (tCSs) in patients with inherited retinal diseases.

**Methods:**

We retrospectively studied patients with RP1L1-associated occult macular dystrophy (OMD), Stargardt disease (STGD), and RP. Photoreceptor-directed tCS directed at L-, M-, S-cones and rods at different temporal frequencies were measured using a four-primary LED-stimulator with an annular test field (2° inner diameter and 12° outer diameter). Mean defects (MDs) were calculated by subtracting sensitivities from age-correlated normal values and averaging defects in frequency ranges where single postreceptoral pathways mediate flicker detection. Each patient was characterized by 6 MD values (one value each for S-cones [SMD] rods [RMD]; two values each for L- [LMD_low/high_] and M-cones [MMD_low/high_], where low refers to 1–6 Hz and high to 8–20 Hz temporal frequency ranges). Groups of similar phenotypes were identified with (supervised) decision trees and (unsupervised) hierarchical classification trees (based on nearest neighbors) and compared with the clinical diagnoses.

**Results:**

The pruned decision tree used RMD for separating RP/STGD from normal/OMD, LMD_low_ for separating OMD from normal, and SMD for discriminating between RP and STGD. The accuracy was 66%. The hierarchical tree (independent of clinical diagnosis) was cut to four clusters, resulting in one cluster containing mainly normal participants, one cluster with severe L- and M-cone defects caused by OMD or STGD, one cluster with severe rod defects (4/5 with RP) and a large cluster with intermediate rod and cone defects that was dominated by RP and STGD patients.

**Conclusions:**

LMD_low_, SMD, and RMD were the most important parameters. Photoreceptor-directed tCSs allow sophisticated functional phenotyping of inherited retinal diseases and complement other structural and functional parameters for genotype–phenotype correlations.

In the last years, immense progress has been made in genetic characterization^1^^,^^2^ and in genotype–phenotype correlations of inherited retinal diseases (IRDs).^3^ Imaging methods like ocular coherence tomography^2^ and adaptive optics scanning laser ophthalmoscopy^4^ allow characterization of structural damage at a microscopic level in vivo and can even demonstrate direct structural correlates of functional processes in photoreceptors (optoretinography).^5^ However, functional studies are still needed, because they allow the characterization of retinal signal processing at the photoreceptoral and the postreceptoral level, and they allow us to characterize the consequences on the patients’ vision.^6^^–^^8^

Techniques that are used currently for characterizing function of photoreceptor subtypes, such as chromatic perimetry^9^ or dark-adapted perimetry,^10^^–^^12^ have several disadvantages. First, the stimuli can lead to responses of multiple photoreceptor types,^6^ especially in the mesopic range. Second, different states of retinal adaptation are used for studying different photoreceptor responses. Different states of adaptation may lead to different relationships between photoreceptor loss and psychophysical thresholds, and this may simulate selective loss.^7^ Finally, the involvement of postreceptoral retinal pathways in currently used perimetry is unclear.^6^

An alternative for isolating photoreceptoral responses is the silent substitution technique. It is not based on retinal adaptation, but rather on changing the spectral composition of a stimulus in a way that changes the excitation in only one photoreceptor subtype.^13^^,^^14^ The use of silent substitution can evade the limitations of the currently used methods. Recently, we studied silent substitution–based photoreceptor-directed temporal contrast sensitivities (tCS) as potential readout parameters in patients with RP,^15^ Stargardt disease (STGD),^16^ and occult macular dystrophy (OMD).^17^

In these studies, a circular, homogeneously illuminated test field covering the perifovea was used. A mesopic mean retinal illuminance (289 phTd) and a whitish mean chromaticity were used.^18^^,^^19^ The intensity of the four LEDs that illuminate this field were sinusoidally modulated over time at temporal frequencies between 1 and 20 Hz and could be detected as periodic changes in the perceived color and/or luminance. The temporal LED contrasts of this modulation were calculated in a way that (1) excitation remained constant in the untargeted photoreceptor types (and for which the modulation was “silent”) and (2) the Michelson contrast of the targeted photoreceptor type was known.^20^^,^^21^ Thus, the perceived changes were driven by only one photoreceptor type.

Specific stimulus characteristics (such as temporal frequency) determine which postreceptoral pathways drive perception.^18^^,^^22^ For understanding our results, it is important to know that the perception of L-cone– and M-cone–directed stimuli is mediated by the red–green opponent system at low temporal frequencies (of which the parvocellular pathway is the physiological basis; the stimulus in the test field is perceived as a chromatic modulation between reddish and greenish) and by the achromatic luminance system at high temporal frequencies (through activity in the magnocellular pathway; the stimulus is perceived as an achromatic flicker).^18^^,^^22^^,^^23^ Importantly, postreceptoral mechanisms also influence how perception of stimuli is altered in patients with IRDs.^6^^–^^8^^,^^24^

In our previous studies, we could describe differences in the tCSs between a specific patient group and normal control participants. However, tCS were not compared between different patient groups and the diagnostic power for differential diagnosis was not examined. The goal of the present study was to find out whether tCS losses directed at different photoreceptor subtypes can identify functional phenotypes as a prerequisite for more fine-grained genotype–phenotype correlations and refined diagnoses in larger cohorts.

## Methods

### Overview of Used Experimental Methods

**Figure 1.**
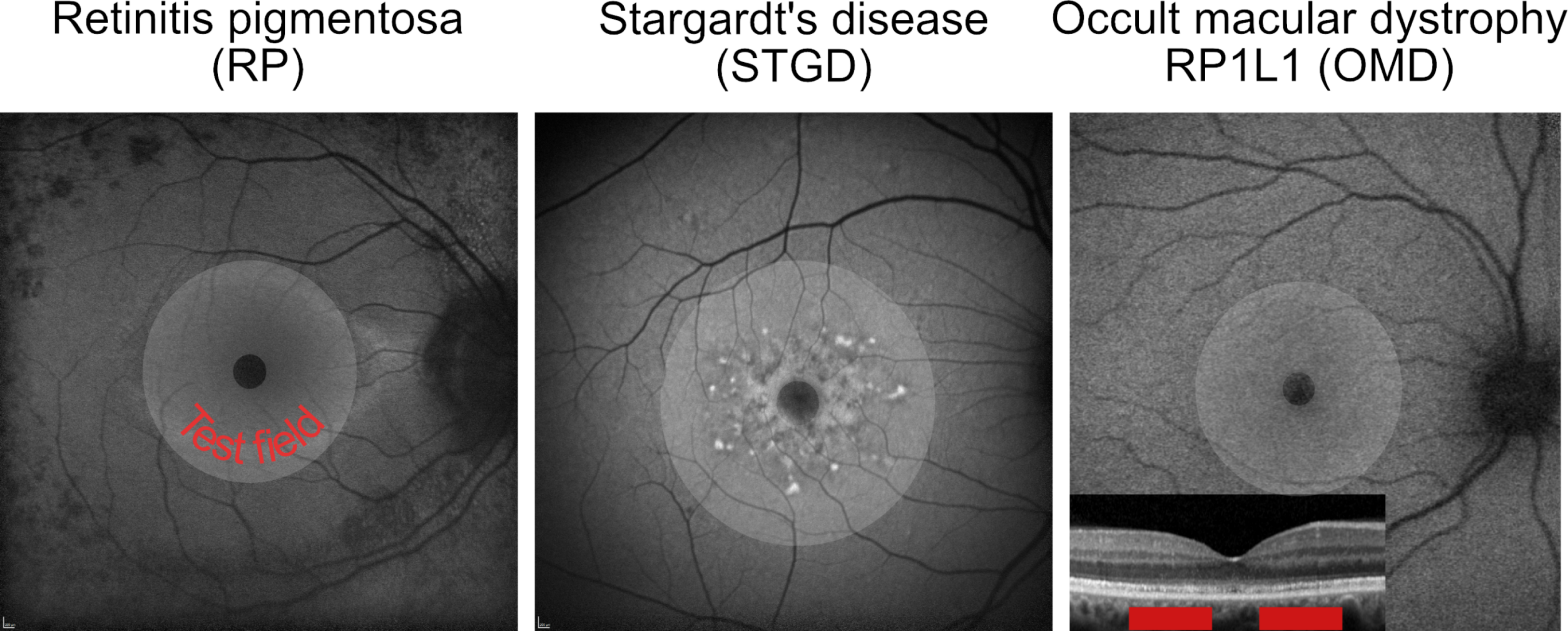
Illustration of the projection of the test field onto the retina in the three disease groups. Genotypically and phenotypically, RP and STGD can be very heterogeneous. Even in patients with OMD caused by the p.R45W mutation in the RP1L1 gene, considerable variability has been reported.

In past studies, we used an apparatus previously described by Pokorny et al.^25^ Briefly, it consists of eight LEDs with four different primary colors (“primaries”: red, emission spectrum maximum at 660 nm; green, 558 nm; cyan, 516 nm; and blue, 460 nm; bandwidths at half height of 8–10 nm) and a Maxwellian View optical pathway, where the spatial structure of the stimulus (central circular field with 2° diameter, surrounded by annular field of 12° outer diameter) is created by an optical cube with masks that is projected onto the retina establishing a center-surround structure. The stimulus geometry is shown in Figure 1.

The image of the LEDs is projected onto the pupillary plane. The LED spectral bandwidths are reduced by interference filters to 8 to 10 nm (full bandwidth at half height) and the intensities of the LEDs are driven by a soundcard.^2^ The advantages of this setup are (1) the high retinal illuminances enabled by the Maxwellian view, (2) the very high temporal resolution enabled by the soundcard (normal displays refresh at rates around 60 Hz and are just barely faster than the visual system whereas the soundcard has a resolution in the kHz range), and (3) the good separation between photoreceptor subtypes owing to the small bandwidths provided by the interference filters. The disadvantages are the fixed spatial structure and the lack of a fifth primary color that would allow controlling the intrinsic–photosensitive retinal ganglion cells.

The silent substitution paradigm allows creating photoreceptor-directed stimuli by varying the spectral composition of the test field.^3^^,^^4^ In practice, the so-called A-matrix is calculated, a square matrix that relates the LED intensities to the relative excitation of the different photoreceptor subtypes.^5^ It is based on the LED spectra and the fundamentals of the different photoreceptors. The LED contrasts necessary to obtain desired photoreceptor contrasts can be calculated by using the inverse of the A-matrix. The advantage of the silent substitution paradigm is the use of identical mean states of adaptation (in terms of luminance and chromaticity) for all photoreceptor types. This is important, because adaptation itself can influence perceptual thresholds significantly.

In our studies, we always used the outer, annular field (2° inner and 12° outer diameter) as the test field and the inner, circular field for fixation. The mean retinal illuminance of the test field was 289 phot Troland when viewed through a 3-mm artificial pupil and resulted in a (reddish–)white background with the CIE coordinates of *x* = 0.38 and *y* = 0.28. The LED intensities were modulated sinusoidally around these settings (i.e., the stimulus was not an increment upon a background). The silent substitution results in modulation of the excitation of only one photoreceptor type around the mean. The threshold excitation modulation of a single photoreceptor type for luminance or color modulation were obtained using a two-interleaved staircase (PEST) procedure at different temporal frequencies. Sensitivities were calculated as the inverse of the photoreceptor contrast (defined as the Michelson contrast of the photoreceptor's excitation modulation) at threshold and plotted as a function of temporal frequency resulting in the tCS curves. In our previous studies, we thoroughly validated these measurements by measuring dichromats and a patient with S-cone monochromacy and by bleaching rods with an intense white light.^19^ Furthermore, we estimated the impact of deviations of the actual observer from the standard observer in terms of prereceptoral filters (lens ageing and macular pigment) and spectral sensitivities of the L- and M-cones owing to gene polymorphisms.^18^^,^^19^

We also published a mathematical model for loss of tCS with ageing based on measurements at 1, 4, 10, and 20 Hz in 20 normal observers between 23 and 83 years of age, as well as full tCS curves (1, 2, 4, 6, 8, 10, 12, 16, and 20 Hz) from 9 of these observers.^7^ These references allow the calculation of mean defect (MD) values, which facilitates the interpretation of sensitivity losses in patients as a function of temporal frequency separately for the different photoreceptor types.

Early results in normal participants showed that thresholds should not only be interpreted in the context of the targeted photoreceptor type, but also in the context of the postreceptoral pathway that mediates perception. Our experiments showed that perception of L-cone– and M-cone–directed stimuli was modulated by the parvocellular system at 2 Hz and by the magnocellular system at 20 Hz.^18^^,^^26^

A relatively heterogenous group of RP patients (*n* = 15) was examined for our first proof-of-concept study.^6^ This study showed that measuring photoreceptor-directed tCS function (and losses) in patients with low vision owing to IRDs is feasible, although the data were noisier than in normal participants, and floor effects frequently occurred. Noise effects could be diminished substantially by averaging temporal sensitivity defects across neighboring frequencies, where perception was mediated by identical postreceptoral pathways.

The present study is based on the results of measurements in three patient groups:

The first study included 15 patients, among whom were also patients with syndromic RP (Usher and neuronal ceroid lipofuscinosis, although the latter was diagnosed after our study) as well as two female carriers and one patient with RPGR-related IRD who had significant cone degeneration. The two female carriers had significant cone damage with visual loss and dyschromatopsia in addition to mild bone spicules, nyctalopia, and constriction of the visual field. Not all RP patients were characterized genetically.^15^ Our study corroborated earlier findings by Seiple et al.^27^ of significant loss of S-cone–mediated function^7^ and showed that rod damage was best demonstrated at higher temporal frequencies around 12 Hz. Also, we found that analysis of L- and M-cone ratios did not suggest alterations in postreceptoral processing.
The second study included 14 patients with genetically confirmed ABCA4-related IRDs (fundus flavimaculatus and Stargardt).^8^ Some patients had hypomorphic variants,^16^ several displayed mild phenotypes, and no patients with ABCA4-associated cone–rod dystrophy were included. Thus, patients were grouped into three groups: (1) fundus flavimaculatus, (2) Bull's eye maculopathy with or without flecks, and (3) central atrophy with eccentric fixation. In contrast with RP, functional loss was more evenly distributed across photoreceptor types. Again, averaging tCS defect values allowed reduction of noise and imputation of missing values.
The third study was on 11 patients with RP1L1-related OMD.^9^ All OMD patients (*n* = 11) had the p.R45W mutation of the *RP1L1* gene.^17^ We found two different functional phenotypes. OMD patients with additional myopia had poorer visual acuity and a different pattern in the tCS functions (band pass pattern). Furthermore, we demonstrated that OMD affects predominantly L- and M-cones, whereas rod-mediated sensitivities were not affected at all.

### Study Design and Participants

This retrospective cross-sectional analysis used data from the three previously published prospective studies described elsewhere in this article (RP,^15^ STGD,^16^ and OMD).^17^ These studies were conducted between 2015 and 2023 and fully adhered to the tenets of the Declaration of Helsinki. All studies were approved by the ethics committee of the medical faculty of the Friedrich Alexander University Erlangen–Nürnberg and all participants gave written informed consent.

Data from one additional RP patient was included, resulting in a total number of 16 patients.

### Clinical Examination

Clinical examinations were carried at in Erlangen and in Tübingen. They included best-corrected visual acuity using Snellen charts, slit-lamp examination, non-contact tonometry, dilated fundus examination, and spectral-domain optical coherence tomography (HRA, Heidelberg Engineering, Heidelberg, Germany). Visual fields were examined with the Octopus 900 perimeter using the M pattern (10°). In some of the RP patients, Goldmann perimetry was performed instead. Color vision was assessed to rule out anomalous trichromacy in all patients, most frequently with the trivector version of the Cambridge Color Test in most patients (Metropsis, Cambridge Research Systems, Cambridge, UK). Sometimes, color vision was examined with the Farnsworth Panel D15 and/or an anomaloscope examination with the HMC anomaloscope (Oculus, Wetzlar, Germany) instead.

### Test Paradigm

The stimulus parameters are described in detail above. Briefly, CIE-1931 coordinates were *x* = 0.38, *y* = 0.28. The retinal illuminances (3-mm pinhole) were 144.5 phot Td in the unmodulated center, and 289 phot Td in the surround field. Fuoll tCS functions (sinusoidal modulation at temporal frequencies of 1, 2, 4, 6, 8, 10, 12, 16, and 20 Hz) were obtained using a staircase algorithm with two randomly interleaved staircases, one starting at maximal and the other at zero contrast.

#### tCS Parameters

Photoreceptor-directed tCSs were defined as the inverse of the Michelson contrast at the photoreceptor level (1/C_threshold_) and converted to decibel (10 * log10(1/C_threshold_)). tCS defects were calculated by subtracting age-correlated normative values^15^ from the measured tCS. Thus, negative defect values indicate tCS loss. When the participant did not perceive changes in the test field at maximal contrast (20%–30% for L- and M-, as well as rods, > 70% for S-cones) this maximal contrast served as a conservative estimate for the C_threshold_.

To reduce noise and to deal with missing values, we averaged tCS losses at certain temporal frequency ranges and called these values tCS MDs.^16^ These ranges also correspond to specific postreceptoral pathways (L- and M-cones: 1, 2, 4, and 6 Hz dominated by the parvocellular, red–green opponency system: LMD_low_/MMD_low_, and 8, 10, 12, and 20 Hz dominated by the magnocellular, achromatic luminance system: LMD_high_/MMD_high_, S-cones: 1, 2, 4, and 6 Hz dominated by the koniocelluar system [SMD]). RMD represents rod-driven defects and was calculated by averaging sensitivities at 6, 8, 10, and 12 Hz.

### Data Analysis

Data were analyzed with the statistical programming language R.^28^ *P* values of less than 0.05 were considered statistically significant. Correction for multiple testing was performed with Holm's method. When several measurements where available for one eye, measurements were averaged. Furthermore, when measurements were available for both eyes, these were also averaged owing to the high symmetry of IRDs.

#### Group Comparisons

The Kruskal–Wallis test was used to identify differences between groups for different parameters. Correction for multiple testing was performed according to Holms. Post hoc testing was performed with Dunn's test. For Supplementary Table S1, Hedge's g statistics were calculated the *cohen.d()* function from the effsize package.^29^

#### Cluster Analysis

The results for each patient comprise six MD values (L-cone MD and M-cone MD, each at low and at high temporal frequencies, S-cone MD and rod MD). Thus, each patient can be represented as a point in a six-dimensional space. Patients who are closer to each other (Euclidean distance) are functionally more similar. These data were analyzed with supervised analysis, where patients are grouped in a way that maximizes concordance with the clinical diagnoses, and by unsupervised analysis, where patients are grouped into clusters of nearest neighbors.

In the supervised analysis, a decision tree is constructed based on the known clinical diagnoses. At each node of the tree, the cohort is divided by a cut-off along one parameter (decision rule). Thus, groups are divided by planes that are perpendicular to one axis in the six-dimensional space. The groups do not necessarily represent clusters that are united by spatial proximity, because points that are very close to each other may fall in different groups. Instead, the algorithm optimizes concordance between the predicted diagnosis and the clinical diagnoses (ground truth). Because too many decision rules in a relatively small dataset can result in overfitting, we reduced the number of decisions (pruning). This process was guided by a complexity parameter and resulted in three rules (along three axes). The complexity parameter represents a threshold for the reduction of error that an additional decision rule must allow for being considered worthwhile. The groups were illustrated in a three-dimensional (3D) space based on the three parameters used for the decision tree.

These analyses were carried at using recursive partitioning with the *rpart()* and *prune()* functions from the rpart package.^30^ A node had to consist of at least five observations for further splitting (*minsplit* parameter). Accuracy was calculated as the number of patients where disease classification was correctly predicted by the tree divided by the number of all patients. Then, complexity was reduced by removing downstream branches that yielded little improvement of accuracy (pruning with a complexity parameter of 0.1). This improved interpretability of the resulting model.

In the unsupervised cluster analysis, points that are close to each other are grouped into small clusters, and then clusters that are close to each other are recursively grouped into larger clusters (hierarchical tree). These clusters do not necessarily correspond with clinical diagnoses. Eventually, all clusters are grouped into one large cluster, but the process can be stopped at an arbitrary number of clusters (cutting). Here, we limited the number of clusters to four, corresponding with the numbers of diagnostic groups.

For illustration purposes, we will also show the resulting clusters in 3D space, where the axes are based on the parameters determined by the supervised cluster analysis, but here, these plots do not contain all information, because the other parameters also go into the distance calculation.

Unsupervised clustering was performed with the *hclust()* and *cut()* functions in R (stats package)^28^ using complete linkage analysis and Euclidean distance. Because all photoreceptor-directed MDs are of similar magnitude and have the same unit, we decided to not perform a normalization. Bootstrap analysis of the cluster stability was carried out using the *clusterboot()* function from the fpc package.^31^ For this analysis, the dataset was resampled with replacement and a similarity of the trees resulting from each resampled dataset were characterized by the Jaccard index. A Jaccard index of greater than 0.6 was considered to indicate sufficient stability.

The supervised cluster analysis is based on the known clinical diagnoses and cuts the space along planes in a way that points that are very close to each other may be in separate groups. The unsupervised cluster analysis is not based on clinical diagnoses, and clusters of points that are very close to each other are separated from the other clusters by greater distances.

## Results

**Figure 2.**
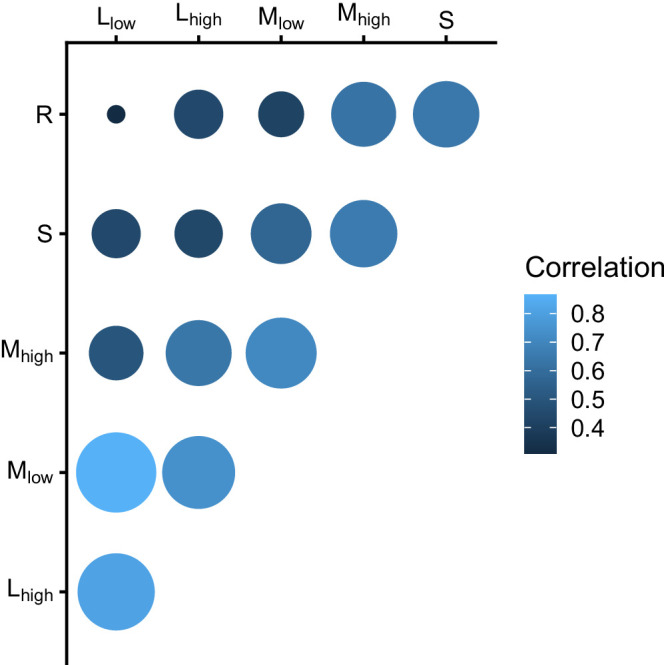
Correlation plot showing the correlation between photoreceptor-related tCS defects (*larger circles* and *bright blue color* indicate high correlation coefficient). The highest correlation can be found between L- and M-cone-driven stimuli at low temporal frequencies. At these low frequencies, the perception of L- and M-cone stimuli is mediated by the parvocellular (red–green opponency) system, which has mechanisms for balancing the inputs of both photoreceptor types. In L- and M-cones, sensitivity defects correlate between high- and low temporal frequencies. Furthermore, S-cone–driven and rod-driven losses are also correlated.

We included 50 participants (normal, 9; RP, 16; STGD, 14; OMD, 11). Both eyes were examined in two patients. Seven eyes were examined twice, six eyes three times, and two eye were examined four times. When several measurements were available, parameters were calculated and then averaged for each patient for cluster analyses. The clinical and demographical characteristics are summarized in Table. Photoreceptor-driven defects directed at different photoreceptors and driven by different pathways are often correlated. This is illustrated in Figure 2.

**Table.** Demographic and Clinical Characteristics

	Normal	RP	STGD	OMD	*P* Value
No.	9	16	14	11	
Age, years	35.33 (14.48)	40.06 (16.40)	47.20 (12.50)	52.45 (14.66)	0.044
Spherical equivalent	−0.96 (1.77)	−3.62 (4.71)	−1.25 (2.35)	−3.18 (4.49)	0.398
LogMAR	0.00 (0.00)	0.27 (0.26)	0.54 (0.46)	0.84 (0.27)	<0.001
Octopus 900 perimetry: MD^*^	0.75 (0.81)	13.41 (8.08)	12.05 (8.98)	6.49 (1.34)	0.010
CCT: Protan axis		11.96 [7.44–17.17]	38.53 [6.25–100.69]	74.41 [34.13–102.72]	0.053
CCT: Tritan axis		71.76 [30.92–85.08]	44.05 [9.80–92.39]	28.17 [15.25–66.47]	0.895

CCT, Cambridge color test.

Values are shown as mean (SD) when normally distributed or as median [interquartile range] when not (only axes from the Cambridge Color Test).

* Positive MD values denote loss of sensitivity.

### Differences Among Groups

**Figure 3.**
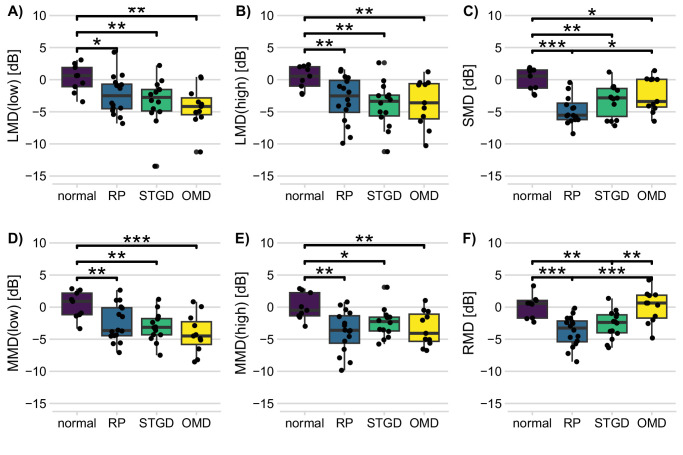
**(A–F)** One-dimensional analysis of photoreceptor-directed tCD parameters. In all tests, all patient groups had lower sensitivities than normal participants, with the notable exception of RMD for the OMD patients. The only differences between patient groups that remained significant after correction for multiple testing were between RP/STGD and OMD for RMD, and between RP and OMD for SMD. Note that there is a bimodal distribution of LMD_low_ and MMD_low_ in RP patients and SMD in OMD patients, of SMD in OMD patients, and of RMD in normal participants. *P < 0.05; **P < 0.01; ***P < 0.001

All types of photoreceptor-directed tCS defects were different between diagnosis groups after Holm correction for multiple testing (Kruskal–Wallis: *P* < 0.05 for LMD_low_, LMD_high_, MMD_low_, and MMD_high_ and *P* < 0.005 for SMD and RMD). In all disease groups, L-cone– and M-cone–driven contrast sensitivities (at low and high temporal frequencies) were poorer than in the control group (Dunn's test) (Fig. 3). However, the differences among patient groups were not significant.

In contrast, S-cone–driven defects were significantly larger in the RP group than in the OMD and control groups. In the STGD group and the OMD group, they were larger than in the control group. Similarly, rod-driven defects were larger in the RP group than in the OMD and control groups. STGD Patients also had larger rod-driven defects than OMD patients and normal participants. The losses in rod-driven sensitivity did not differ between RP and STGD patients.

Although the group differences are highly significant, there is a certain amount of overlap. We calculated the effect sizes (Hedge's g statistic), which can be used for power calculations in future studies. They are shown in Supplementary Table S1.

### Supervised Cluster Analysis

**Figure 4.**
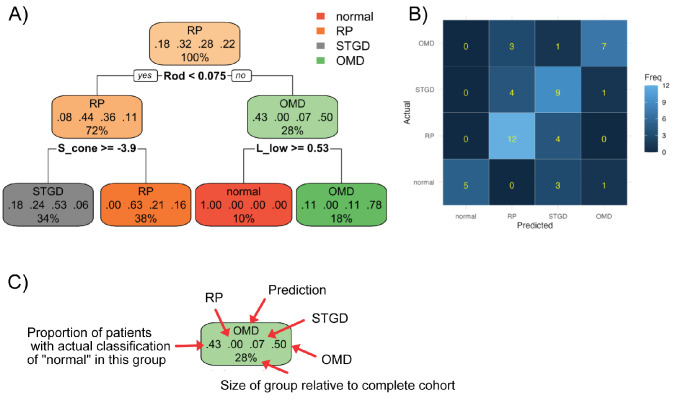
**(** **A)** Pruned classification tree. The process of pruning removes those branches from the tree that do not improve that classification much. The resulting decision tree is less likely to represent an overfit. Each node is represented by a box and classified by the most likely diagnosis at this point in the tree (*top*). The four numbers in the *middle* show the proportion of the four clinical diagnoses in this node (from *left to right*, normal, RP, STGD, and OMD), and the percentage of all patients that are included in this node. The left branch is followed when the decision rule holds true, the right branch when it does not. The tree should be interpreted as follows: If there is no information on sensitivities at all, assume RP, because this is the most frequent diagnosis in the whole training dataset (32% of patients). If RMD is low (<0.075dB, i.e., poor rod function), assume RP (44% of these patients). Then, if SMD is not very low (≥ −3.9dB), assume STGD (53%), otherwise assume RP (63%). In contrast, if rod function is good, assume OMD (50% of patients), unless red–green color discrimination is good (LMD_low_ ≥ 0.53 dB). In this case, assume normal. **(B)** The confusion matrix for the resulting classification. **(C)** Illustration of the meaning of the numbers in the boxes of the classification tree.

The original classification tree had an accuracy of 86% (95% confidence interval, 73%–94%). However, the resulting decision tree is complex, difficult to interpret, and probably also overfitting the data. Therefore, splits that do not improve the model much were removed until the complexity parameter was less than 0.1 (pruning). The resulting classification tree (Fig. 4) used RMD to distinguish normal/OMD from RP/STD, and then LMD_low_ to distinguish normal participants from OMD patients, and SMD to differentiate RP and STGD. On average, RP patients had more S-cone–driven defects. These rules seemed to be meaningful, interpretable, and adequate for the small number of patient and the four disease groups.

**Figure 5.**
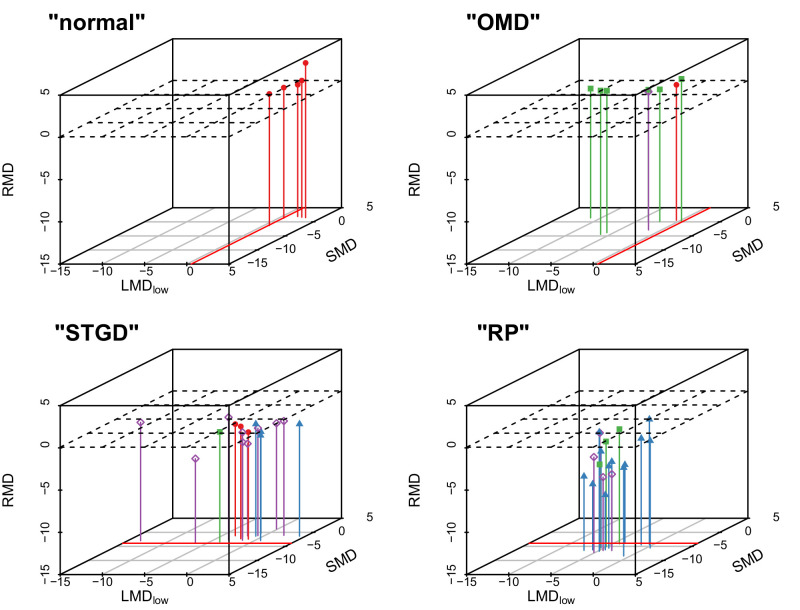
Illustration of predicted disease groups in 3D space based on the parameters identified in the decision tree (supervised classification). The *right upper back corner* corresponds with normal function. The four cubes represent four decision tree predictions (hence the quotation marks), the shape and color of the points represent the true clinical classification (*red dots*, normal participants; *blue triangles*: RP; *purple diamonds*:, STGD; *green rectangles*, OMD). The RMD cut-off used by the decision tree to discriminate normal/OMD from RP/STGD is represented by the horizontal plane, the LMD_low_ and SMD cutoffs are represented by red lines. This is a graphical representation of the classification tree in Figure 4. If RMD is good (i.e., the points are located above the horizontal plane), the algorithm assumes that participants are normal or have OMD; otherwise, it assumes that they have RP or STGD. In the first case, participants are classified as normal if they additionally have a good L-cone–driven function at low temporal frequencies (LMD_low_ ≥ 0.53 dB as indicated by the red lines in the upper panels) and as OMD patients if that is not the case (LMD_low_ < 0.53 dB). In the second case, participants are classified as STGD when S-cone–driven function is relatively good (SMD ≥ −3.9 dB; indicated by the *red lines* in the *bottom*), and as RP if this is not the case.

The confusion matrix is shown in Figure 4. The accuracy of the classification with the pruned tree (number of correctly classified patients/total number) is 66% (95% confidence interval, 51%–79%). The highest frequency of misclassification was between RP and STGD (Fig. 5).

### Unsupervised Cluster Analysis

**Figure 6.**
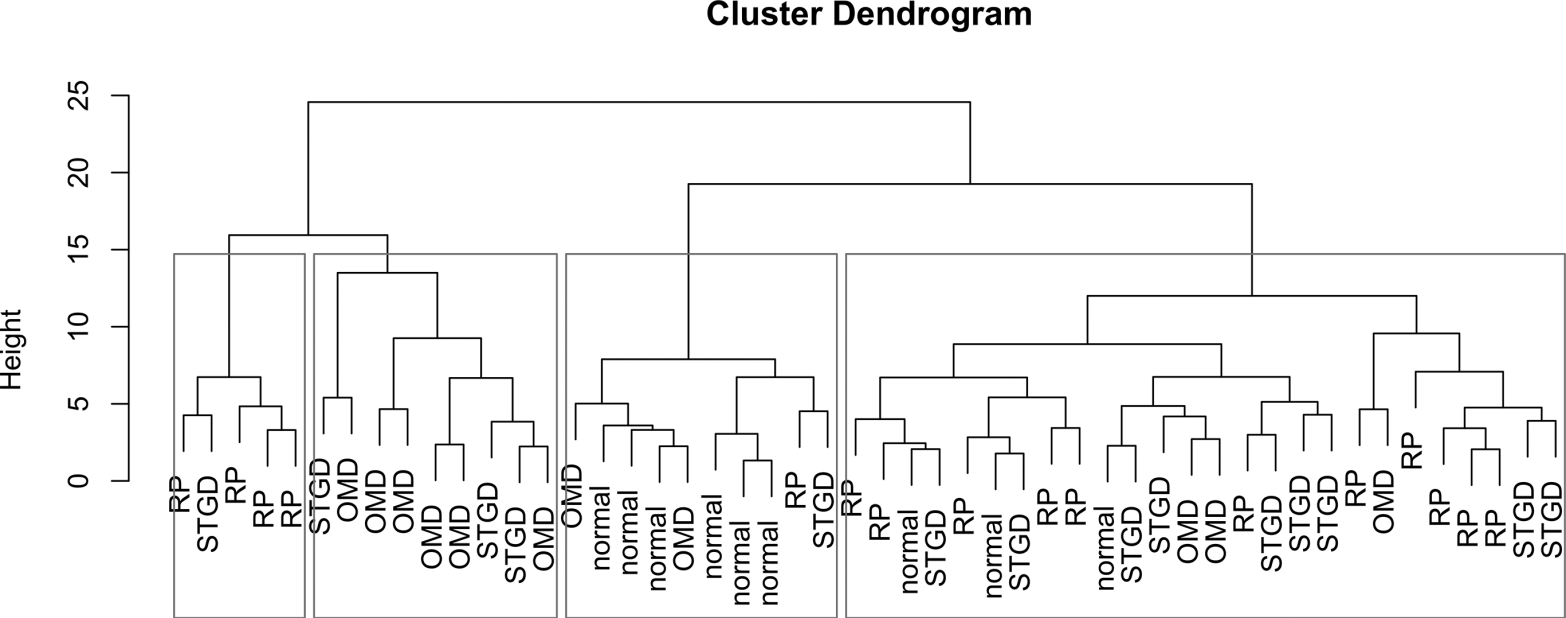
Dendrogram showing the results of the unsupervised cluster analysis. In this type of unsupervised clustering, patients who have very similar patterns in the photoreceptor-directed tCS defects are grouped in clusters. Then, small cluster that are very similar are recursively combined to larger clusters. The height (*y* axis) is a measure of the dissimilarity of the clusters. The higher the merges of two cluster, the more dissimilar are the patients that go into the resulting clusters. The tree can be cut at different heights depending on the number of clusters that are suspected. Here, it was cut at a height of 15, resulting in four clusters (*rectangles boxes*).

Hierarchical clustering was performed using Euclidean distance and the complete linkage method. The dendrogram and the branches that correspond to four clusters are shown in Figure 6. One cluster is dominated by normal participants (cluster 1), one is dominated by OMD patients (cluster 2), and a third, small cluster is dominated by RP patients (cluster 4). The largest cluster contains most STGD patients but is very heterogeneous (cluster 3). Cluster 2 (OMD) comprises two patients with very poor color discrimination and very low LMD and MMD at low temporal frequencies (LMD_low_ < −10 dB). We observed this phenotype previously in dichromats. These two patients would make up a fifth cluster if the tree was cut one level deeper.

When using the most frequent diagnosis in a cluster as a prediction (cluster 1, normal; cluster 2, OMD; cluster 3, STGD; and cluster 4, RP), the accuracy of the resulting classification was 50% (95% confidence interval, 0.36–0.64). Bootstrap testing of the cluster stability showed a small Jaccard index for the two smallest clusters: 2 (OMD) and 4 (RP). This result indicates that these clusters were not very stable and changed in resampled datasets. In contrast, the normal group and the STGD clusters had a Jaccard index 0.73 and 0.64, respectively, indicating good stability.

The patients in cluster 4 (RP) had very poor rod function (low RMD) and very poor red–green discrimination (low LMD_low_). Three patients had RPGR mutations (RP group, two of them very heterozygous female carriers) and one patient had an ABCA4 mutation.

**Figure 7.**
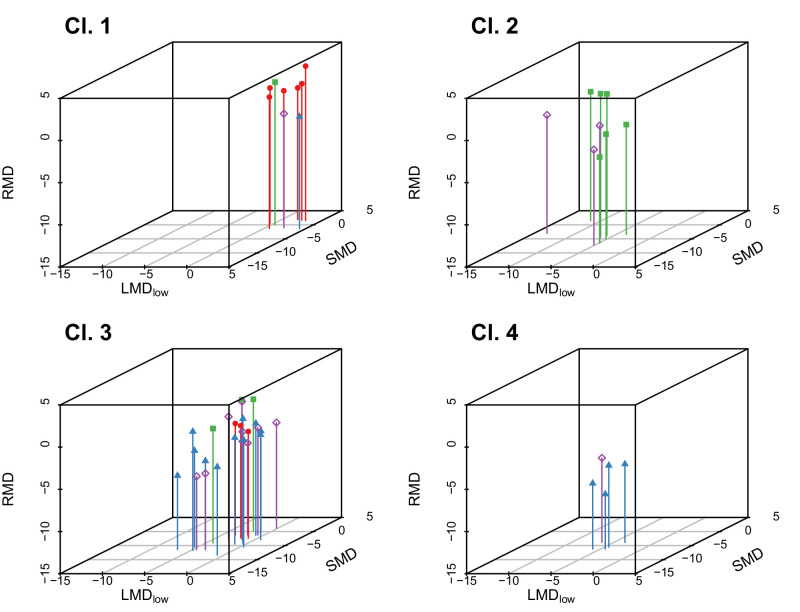
Illustration of four different clusters (1–4) from unsupervised analysis in 3D space. The *right upper back corner* corresponds with normal function. These clusters do not directly correspond with clinical diagnoses, because they are solely based on spatial proximity in the feature space. Cluster 1 is dominated by normal participants, cluster 2 by OMD patients, and cluster 4 by RP patients. Cluster 3 is heterogeneous and comprises participants from all clinical groups, but it contains most ABCA4 patients. *Red dots*, normal participants; *blue triangles*, RP; *purple diamonds*, STGD; *green rectangles*, OMD.

In Figure 7, these clusters are illustrated in 3D space using the axes from the supervised analysis (LMD_low_, RMD, and SMD). Although the clusters are determined in 6D space, the resultant clusters can already be observed in this 3D space, indicating that these three values already contain most of the information.

## Discussion

Perifoveal photoreceptor-directed tCS measurements allowed identification of clusters of functional phenotypes. These functional phenotypes overlapped with the clinical diagnoses but were not identical.

The supervised approach ended up using three parameters: (1) LMD_low_, which relates to red–green color discrimination, (2) RMD, and (3) SMD. Of these parameters, LMD_low_ and RMD are probably directly related to underlying molecular mechanism. In contrast, SMD, which was used for separating RP from STGD, might be related to the topographic distribution of photoreceptor damage within the perifovea.

Early loss of red–green color discrimination is associated with cone dystrophies, including OMD. Note that some diseases from the RP spectrum, for example, those related to RPGR mutations, have early cone involvement. Furthermore, some patients with OMD had relatively good function in the periphery of the perifoveal test field. This explains why low-frequency L-cone–driven tCS (and color vision) was relatively normal despite considerable loss of visual acuity even though both parameters are driven by the same photoreceptors and postreceptoral pathways.

Rod dysfunction is a hallmark of rod (cone) dystrophies. However, some subtypes of RP have normal rods in the central retina,^32^ often surrounded by a hyperfluorescent ring.^33^ In contrast, macular degenerations that involve the RPE have significant loss of rod function, and this includes not only AMD,^34^ but also STGD.^10^^,^^35^

Impaired S-cone function has been observed frequently in all kinds of retinal disease, including RP.^36^ The hypothesis that the S-cones are generally more fragile has been challenged by authors who stressed that S-cones are scarcer, and that, as a consequence of this and other factors, S-cones have a limited dynamic range.^37^ These mechanisms may explain why S-cone dysfunction may be visible earlier in the course of the disease. In this study, SMD values were smaller in RP than in STGD patients. In contrast with ERG, psychophysical thresholds do not integrate responses across the stimulated retina but are rather determined by regional sensitivity.^38^^,^^39^ We tentatively propose that circular retinal degeneration that covers the more eccentric perifovea strongly affects S-cone function. In contrast, many STGD patients in our cohort had hypomorphic alleles and relatively mild phenotypes,^16^ having either a bull's eye phenotype or a central atrophy, leaving the more eccentric perifovea with a relatively high density of S-cones intact. This factor explains why the SMD is relatively small in STGD patients (Fig. 3C).

Finally, there is phenotypic variability even in the genetically homogenous OMD group,^17^^,^^40^^–^^42^ but even more in the STGD group and the genetically very heterogenous RP group. For example, close inspection of Figure 3A shows that LMD_low_ shows a biphasic distribution in the RP group. Some patients have considerable problems in red–green discrimination, including those with RPGR mutations, whereas other patients have relatively well-preserved L-low sensitivity. However, our sample size is too small for analyzing RP and STGD subgroups in our study.

This discussion explains why our disease clusters cannot be identical to the clinical disease classification, which include additional information about the peripheral retina, visual acuity, and retinal structure. However, the accuracies that we observed are quite promising, suggesting that photoreceptor-directed tCS complement other clinical measurements and provide a more complete description of the functional phenotype. This can be used for more sophisticated genotype–phenotype correlations and for more personalized counselling of patients.

### Limitations

Our cohort is relatively small, especially when the genotypic and phenotypic heterogeneity of the RP and the STGD groups are considered, showing different extent of rod and cone degeneration inside of one phenotype group (especially STDG and RP). Overfitting may occur, but we tried to limit the risk by pruning the supervised classification tree and by performing bootstrap analysis for the unsupervised cluster analyses. This study justifies further research in genotypically more homogeneous and structurally better characterized cohorts.

The fixed stimulus geometry with a relatively large test field limits is a further limitation. A silent substitution perimetry would allow measurements in specific regions of interest, for example, before treatment. Potentially, a modified video projector system with four or five primaries could allow more targeted examinations.

Interindividual variability of prereceptoral filters such as the lens or the macular pigment, as well as variability in photoreceptor spectral sensitivities may lead to suboptimal isolation of the targeted photoreceptors in some patients. However, the influence of these factors on the quality of isolation can be estimated quantitatively during the calculations of the silent substitution stimuli, which makes the risk of such errors visible to the investigators. Other psychophysical measurements like chromatic perimetry and dark-adapted perimetry are influenced by these factors to the same degree, but investigators may be oblivious of that risk.

Finally, the dynamic range of the photoreceptor-directed is limited to maximally 12 dB. Floor effects may occur, when patients cannot perceive the maximal possible photoreceptor modulation. In these cases, we used the maximal possible contrast as a conservative estimate for threshold. However, it would be desirable to improve dynamic range in the future.

### Implications for Clinicians

Perifoveal tCSs can be measured in patients with intermediate (to advanced) visual disabilities. Sometimes, patients with a different structural phenotype may have similar functional phenotype in these perifoveal measurements. These overlaps between clusters may be unexpected. For example, one would expect a large difference in rod function between RP and STGD. Partially, the overlap is a consequence of the heterogeneous cohorts. However, stimulus geometry and location in the perifovea are also responsible. For example, rod function may be affected minimally in the central retina,^32^ but the same area may be affected severely in STGD. Furthermore, psychophysical thresholds do not represent a sum response like ERG amplitudes but may be driven by a small area of intact retina in the stimulus area.

Instruments will have to be improved, and measurement protocols will have to be clarified, for more widespread application of these techniques in clinical research. Currently, we are establishing a technique to map photoreceptor-directed tCS across different locations the visual field (silent substitution campimetry).

## Conclusions

Photoreceptor-directed tCS measurements based on the silent substitution technique allow sophisticated functional phenotyping of patients with IRDs.

## Supplementary Material

Supplement 1
