# Supplementary material for: Multidimensional Functional Phenotyping Based on Photoreceptor-Directed Temporal Contrast Sensitivity Defects in Inherited Retinal Diseases
Source: Invest Ophthalmol Vis Sci. 2025 Apr 10;66(4):25. doi: 10.1167/iovs.66.4.25 (PMC11993126; doi:10.1167/iovs.66.4.25)
Supplement: Supplement 1 [file iovs-66-4-25_s001.pdf]

Table S1. Effect size estimations (Hedge's g) for the effects of the three clinical phenotypes on the 6 types of photoreceptor-directed temporal contrast sensitivities (for LMD and MMD, low refers to temporal frequencies of 1-6Hz, whereas high refers to 8- 20Hz). These can be used for power calculations in future studies.

	OMD	RP	STGD
LMD_{high}	1.26	1.06	1.39
LMD_{low}	1.50	0.92	1.07
MMD_{high}	1.33	1.30	1.10
MMD_{low}	1.60	1.05	1.46
RMD	0.03	1.74	1.37
SMD	0.95	2.19	1.38

LMD: M-cone-driven mean tCS defects; MMD: M-cone-driven defects; RMD: rod-driven defects; SMD: S-cone-driven defects.
